# Supplementary material for: A Van der Waals Material Exhibiting Room Temperature Broken Inversion Symmetry with Ferroelectricity
Source: Adv Sci (Weinh). 2025 Dec 5;13(9):e18784. doi: 10.1002/advs.202518784 (PMC12904008; doi:10.1002/advs.202518784)
Supplement: Supplementary file 1 — Supporting Information [file ADVS-13-e18784-s001.pdf]

Supporting Information

Indium Flux Ratio (cm^2s^{-1})	Selenium Flux Ratio (cm^2s^{-1})	Flux Ratio	Substrate Temperature (°C)	Deposition Time (min)
1.20×10^{13}	1.97×10^{14}	16.5	700	60

(a)

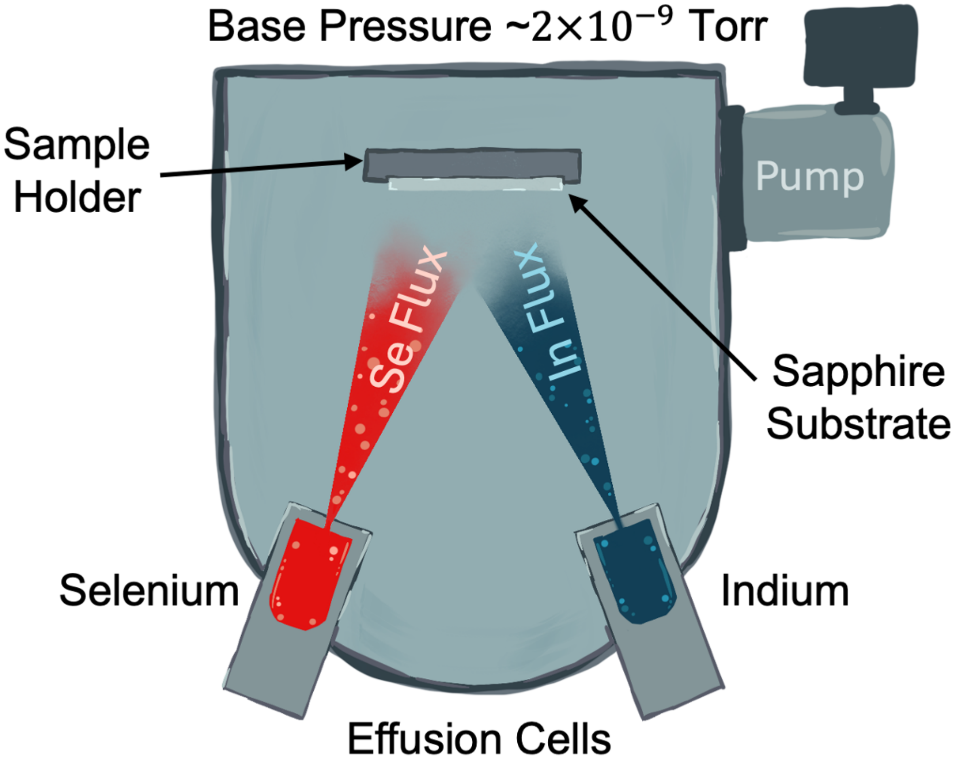

(b)

Figure S1: (a) Detailed synthesis process and growth conditions for large-area optimized indium selenide films deposited on sapphire substrates using MBE. (b) Schematic representation of the MBE setup employed for the deposition, highlighting the key components and configuration.

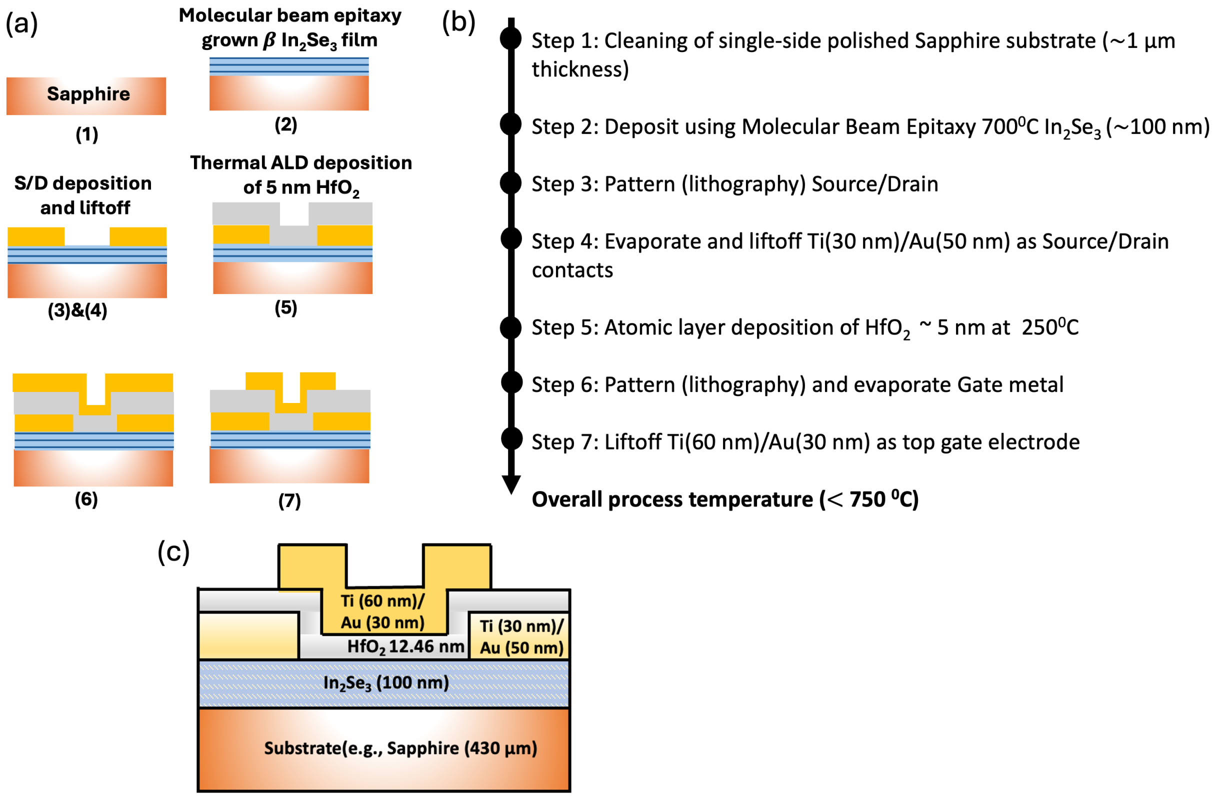

Figure S2: (a) Process flow diagram for the fabrication of the thin-film FET device, detailing each step. (b) Schematic of the fabricated device illustrating the layer dimensions. The entire fabrication process was conducted at temperatures below 700 °C.

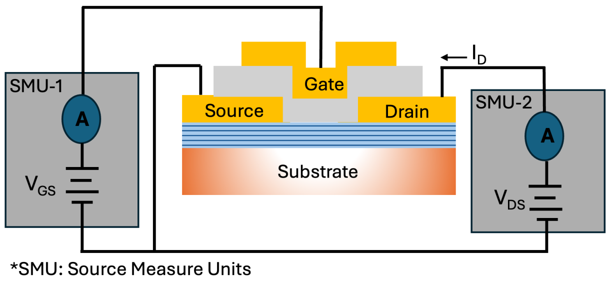

Figure S3: Electrical testing setup and connections. Electrical measurements were performed using two Source Measurement Units (SMUs) as part of a Keithley 4200 Semiconductor Characterization System. A drain-source voltage (V_{DS}) was applied between the drain and source terminals, while the gate terminal was independently biased with a gate-source voltage (V_{GS}).

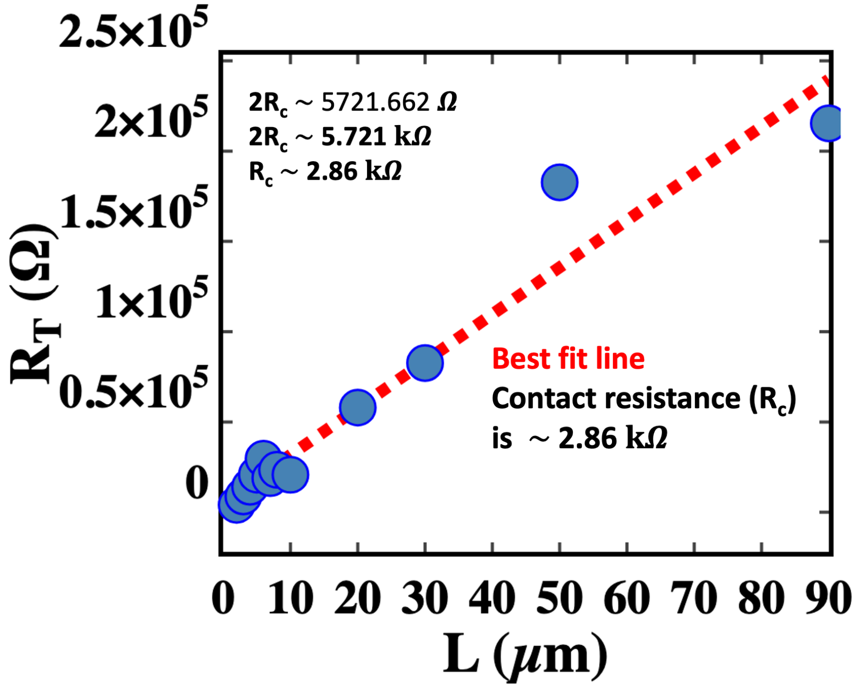

Figure S4: Measurement of R_C using TLM. The channel lengths used in the measurement ranged from 2 μm to 90 μm, specifically: 2 μm, 3 μm, 4 μm, 5 μm, 6 μm, 7 μm, 8 μm, 10 μm, 20 μm, 30 μm, 50 μm, and 90 μm, with a constant channel width of 100 μm. The applied drain bias (V_{DS}) was 2 V, and the total resistance (R_T) was measured at this bias. From the TLM analysis, the R_C was determined to be approximately 2.86 kΩ.

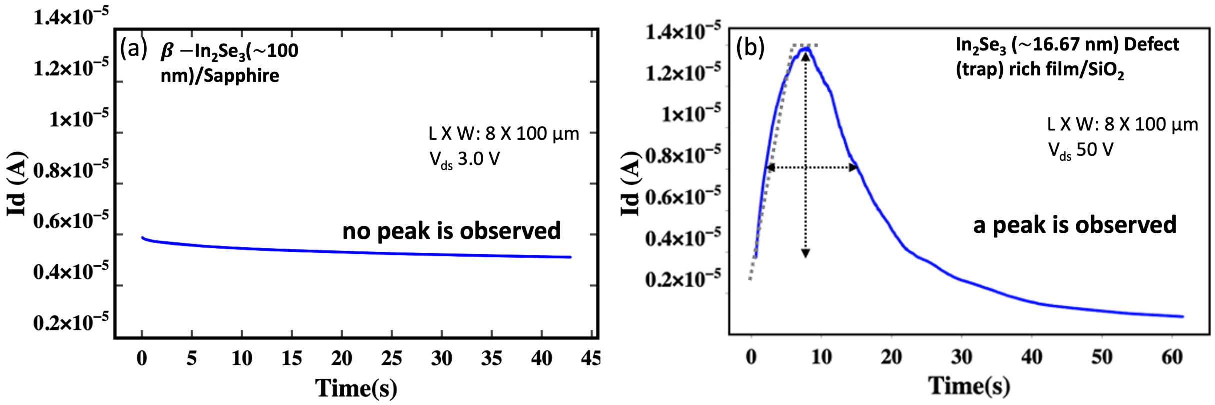

Figure S5: Transient current response analysis conducted on (a) a nominally β-In_2Se_3 film and (b) a defect-rich In_2Se_3 film. No peak is observed in the nominally β-In_2Se_3 film, while a pronounced peak is observed in the trap-rich film, further supporting the conclusion that the observed hysteresis arises from polarization rather than traps.

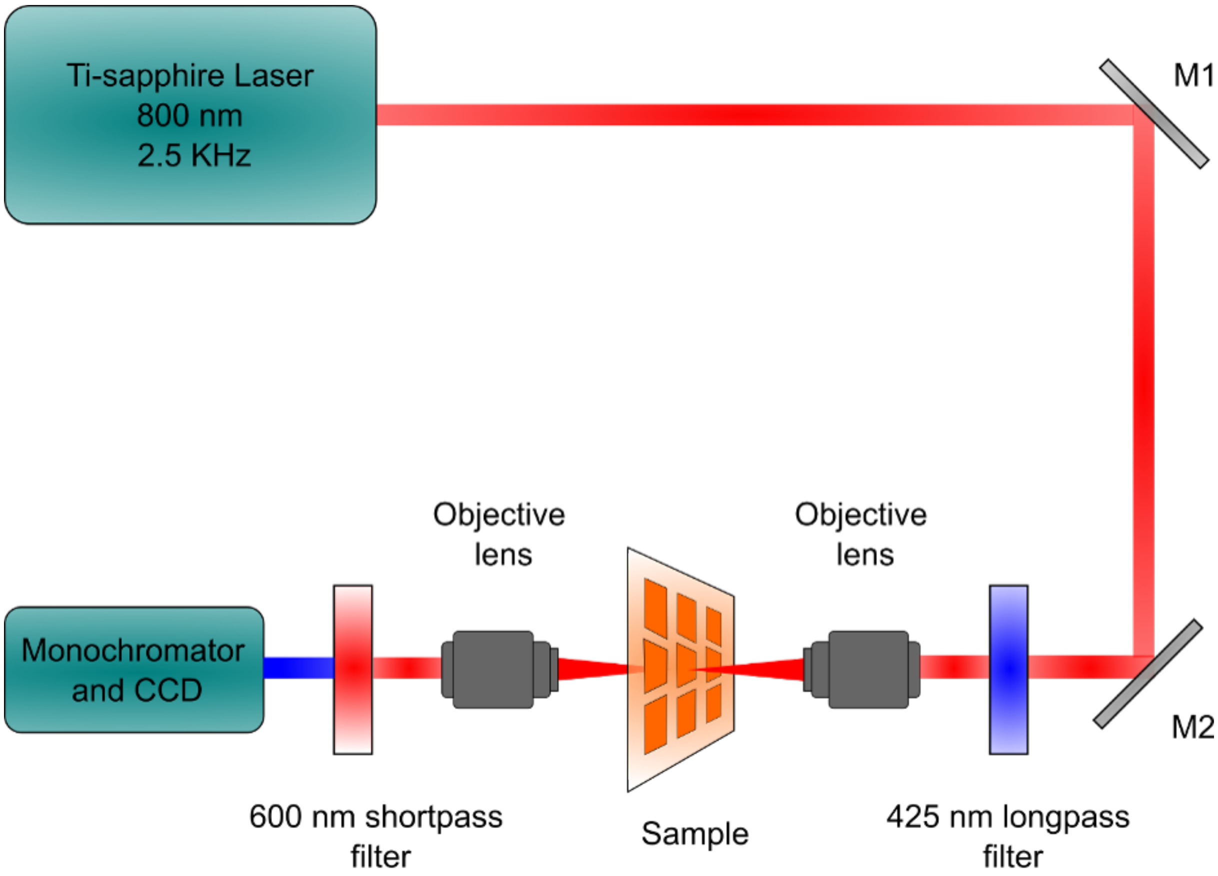

Figure S6: A schematic of the SHG measurement setup. The fundamental light at 800 nm is generated by a Ti: sapphire pulsed laser with a repetition rate of 2.5 kHz. Mirrors M1 and M2 direct the laser light to the sample. A 425 nm longpass filter removes any second harmonic signal originating from the laser itself. The first objective focuses the light on the sample, while the second objective, positioned after the sample, collects the transmitted light. A 600 nm shortpass filter blocks the fundamental laser light, allowing only the second harmonic signal to pass. Finally, a monochromator separates the transmitted light into its spectral components, and a CCD detector records the intensity of each component.

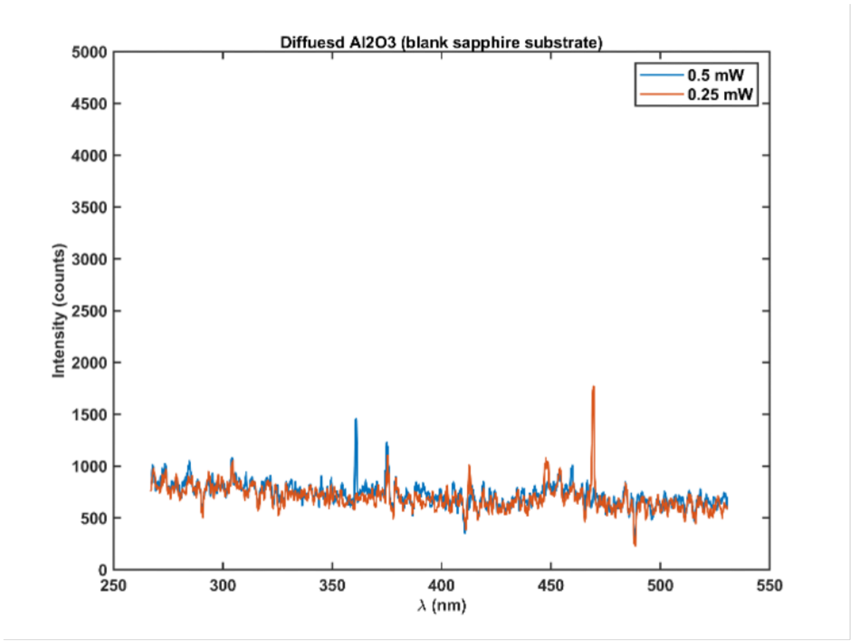

(a) .

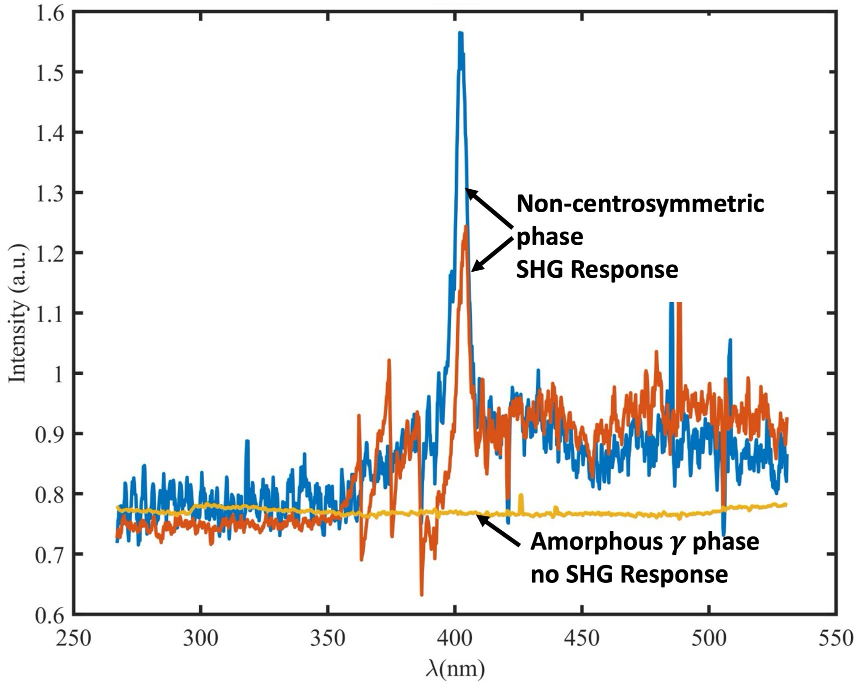

(b) .

Figure S7: SHG measurements for (a) blank sapphire substrates and (b) amorphous γ-phase In_2Se_3 films. Both samples exhibited no detectable SHG response, indicating the absence of noncentrosymmetric properties.

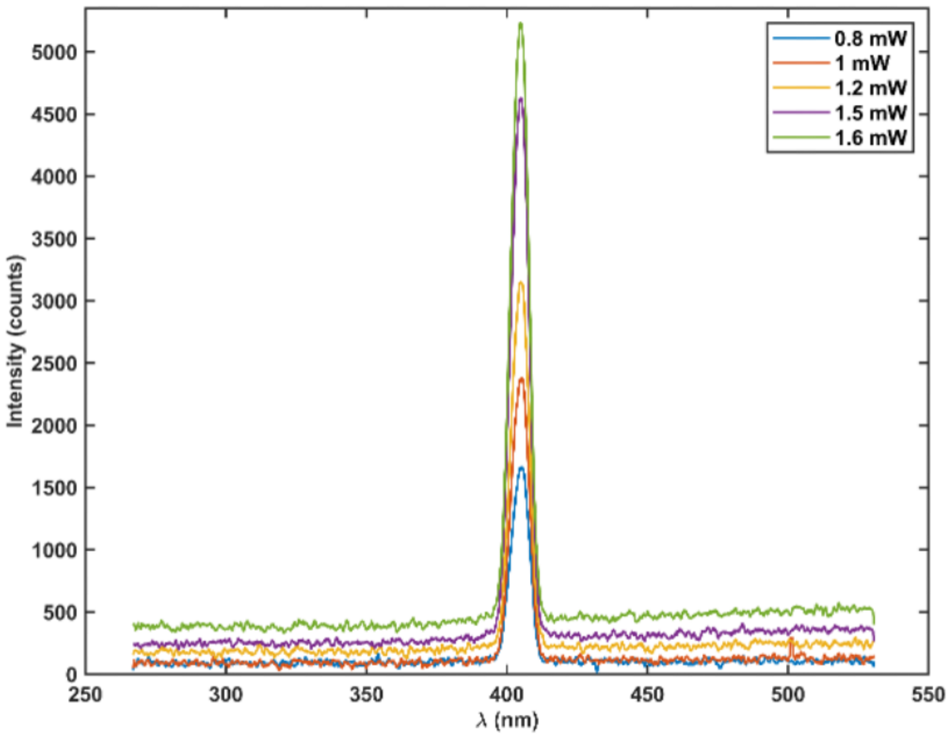

Figure S8: SHG measurements on optimized indium selenide films conducted under varying incident power levels (0.8, 1.0, 1.2, 1.5, and 1.6 mW) of the fundamental light. These measurements were performed on a distinct batch of films to evaluate the SHG response across different power conditions.

Pristine sample		Biased sample		β^p region biased vs pristine
# β^p atoms/nm^2	β^p atomic %	# β^p atoms/nm^2	β^p atomic %	
0.122	0.830%	2.01	13.6%	16×

Table S1: Atomic density and atomic percent of the β^p phase region in pristine and biased samples, showing a 16× increase of the β^p phase in the biased sample.

Pristine sample					Biased sample					β^p region biased vs. pristine
# β^p units	# replaced atoms	Total measured (nm^2)	# β^p atoms/nm^2	β^p atomic %	# β^p units	# replaced atoms	Total measured (nm^2)	# β^p atoms/nm^2	β^p atomic %	
83	415 (83 × 5)	3408	0.122 $\left(\frac{415}{3408}\right)$	0.830% $(\frac{0.122}{14.7}$ × 100%)	1375	6875 (1375 × 5)	3424	2.01 $\left(\frac{6875}{3424}\right)$	13.6% $(\frac{2.01}{14.7}$ × 100%)	16 × $\left(\frac{13.6}{0.830}\right)$

Table S2: Detailed quantification of the β^p phase region in pristine and biased samples. Columns for each sample show: number of β^p units, raw count of replaced atoms, total measured area, computed density, and atomic percentage; the final column gives the ratio of biased vs. pristine.

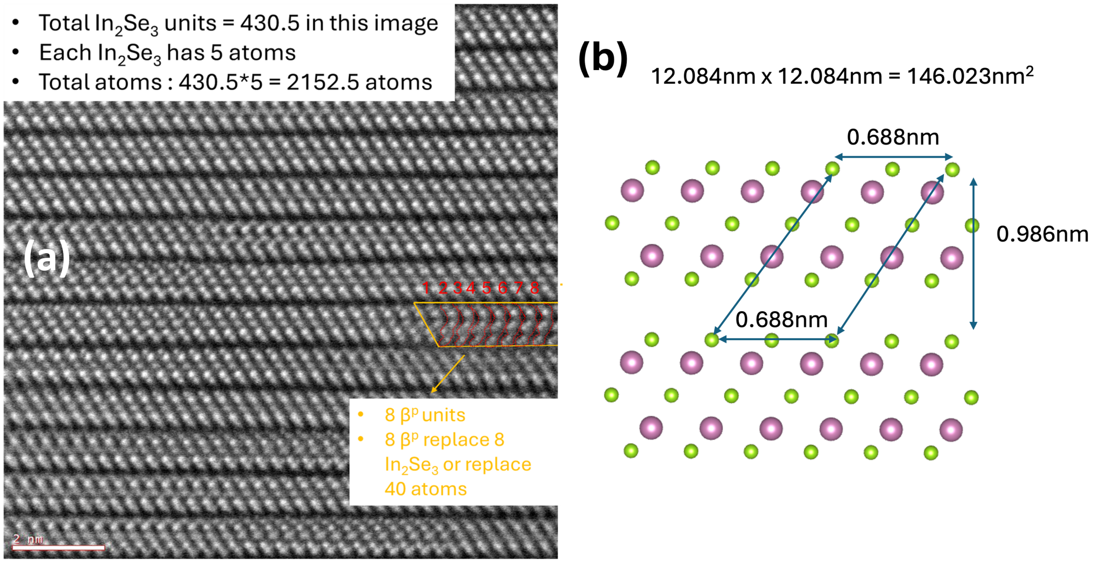

Figure S9: The image dimensions are 12.084 nm × 12.084 nm = 146.023 nm^2. In the parallelogram in b, there are 2 units of In_2Se_3. The area of the parallelogram is 0.688 nm $\times$ 0.986 nm $=$ 0.6784 nm^2. Therefore, the areal density of In_2Se_3 is $\frac{2}{0.6784} = 2.9483$ $In_2Se_3/$ nm^2, which means there are approximately 2.9483 In_2Se_3 units per square nanometer. Assuming each unit contains 5 atoms, this corresponds to $2.9483 \times 5 = 14.742$ atoms$/$nm^2. The total number of In_2Se_3 units in the image is $146.023 \times 2.9483 = 430.5$. Out of these, 8 (inside the orange box) are β^p units. Therefore, the concentration of β^p configurations in this image is approximately 1.85% (8/430.5).

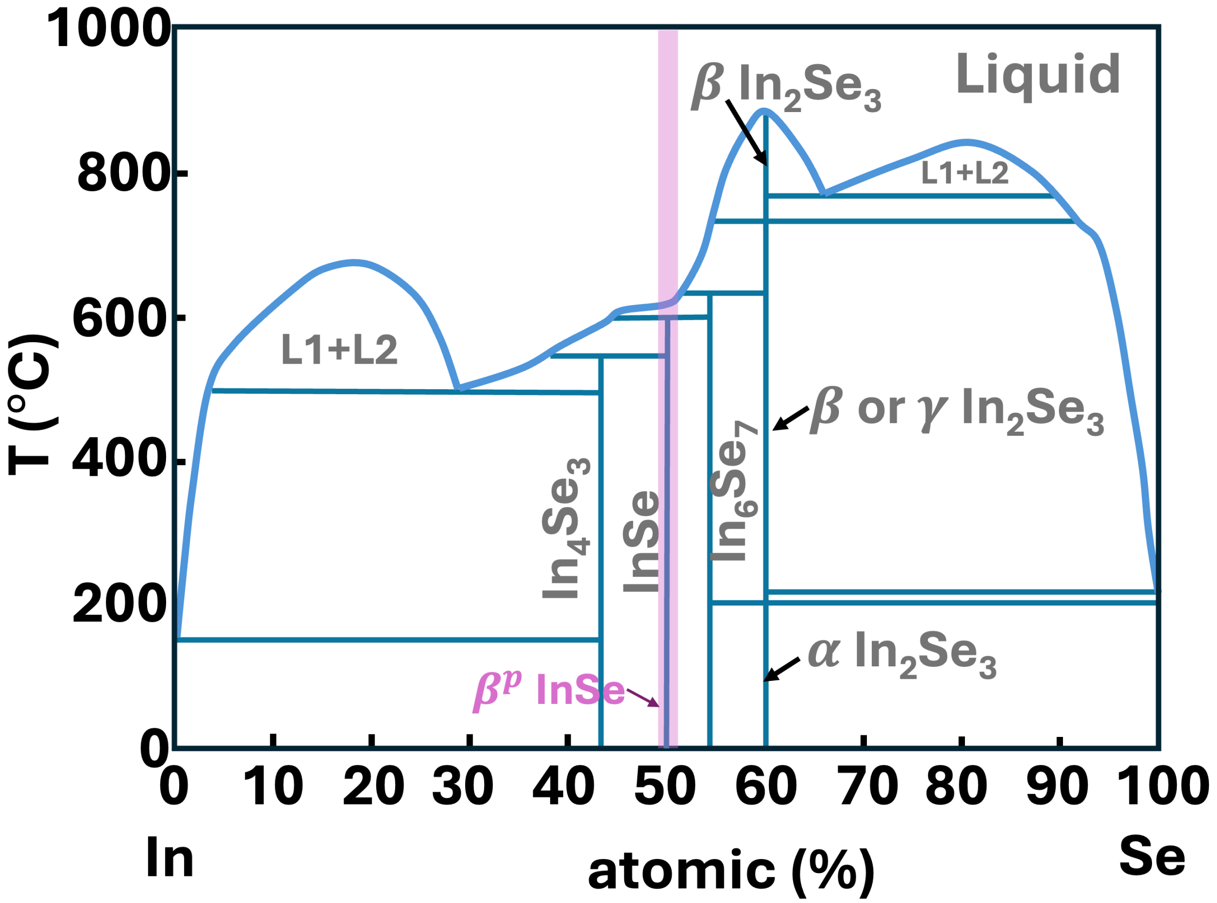

Figure S10: The phase diagram (adapted from (*42*)) shows the predicted phases at the approximate composition. The β^p phase of indium selenide observed at a 50% atomic ratio (purple shaded region) was previously unreported.

**Reference**	**Material**	**Phase**	**Stacking Diagram**
**This work**	**InSe**	β^{p}	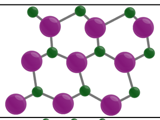
(*43*)	InSe	β	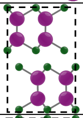
(*43*)	InSe	γ	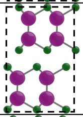
(*43*)	InSe	ϵ	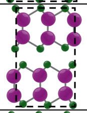
(*43*), (*44*)	In_2Se_3	α - $2H$	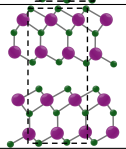
(*43*), (*44*)	In_2Se_3	α - $3R$	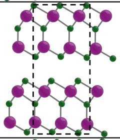
(*43*), (*44*)	In_2Se_3	β - $2H$	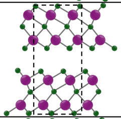
(*44*)	In_2Se_3	β - $3R$	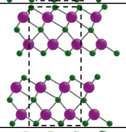
(*44*)	In_2Se_3	β - β'	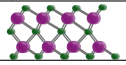
Diagram adapted from Jeengar *et al.* (*45*)	In_2Se_3	γ (3D-structure)	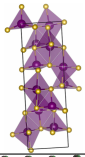
(*44*)	In_2Se_3	δ - $2H$	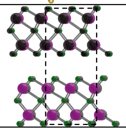
(*46*)	In_2Se_3	κ	No publicly accepted structure, but closely linked to the structure of α

Table S3: Our synthesized β^{p} phase of indium selenide is distinct from all previously reported phases of InSe and In_2Se_3. Purple and green atoms represent indium and selenium, respectively. Yellow indicates selenium in the γ structure.
